# The Ambivalent Function of YAP in Apoptosis and Cancer

**DOI:** 10.3390/ijms19123770

**Published:** 2018-11-27

**Authors:** Xianbin Zhang, Ahmed Abdelrahman, Brigitte Vollmar, Dietmar Zechner

**Affiliations:** Institute for Experimental Surgery, Rostock University Medical Center, Schillingallee 69a, 18059 Rostock, Germany; ahmed.abdelrahman@med.uni-rostock.de (A.A.); brigitte.vollmar@uni-rostock.de (B.V.); dietmar.zechner@uni-rostock.de (D.Z.)

**Keywords:** Hippo, YAP, signaling pathway, cell death, autophagy, p73, cancer, therapy

## Abstract

Yes-associated protein, a core regulator of the Hippo-YAP signaling pathway, plays a vital role in inhibiting apoptosis. Thus, several studies and reviews suggest that yes-associated protein is a good target for treating cancer. Unfortunately, more and more evidence demonstrates that this protein is also an essential contributor of p73-mediated apoptosis. This questions the concept that yes-associated protein is always a good target for developing novel anti-cancer drugs. Thus, the aim of this review was to evaluate the clinical relevance of yes-associated protein for cancer pathophysiology. This review also summarized the molecules, processes and drugs, which regulate Hippo-YAP signaling and discusses their effect on apoptosis. In addition, issues are defined, which should be addressed in the future in order to provide a solid basis for targeting the Hippo-YAP signaling pathway in clinical trials.

## 1. Introduction to YAP and the Hippo Signaling Pathway

**Figure 1:**
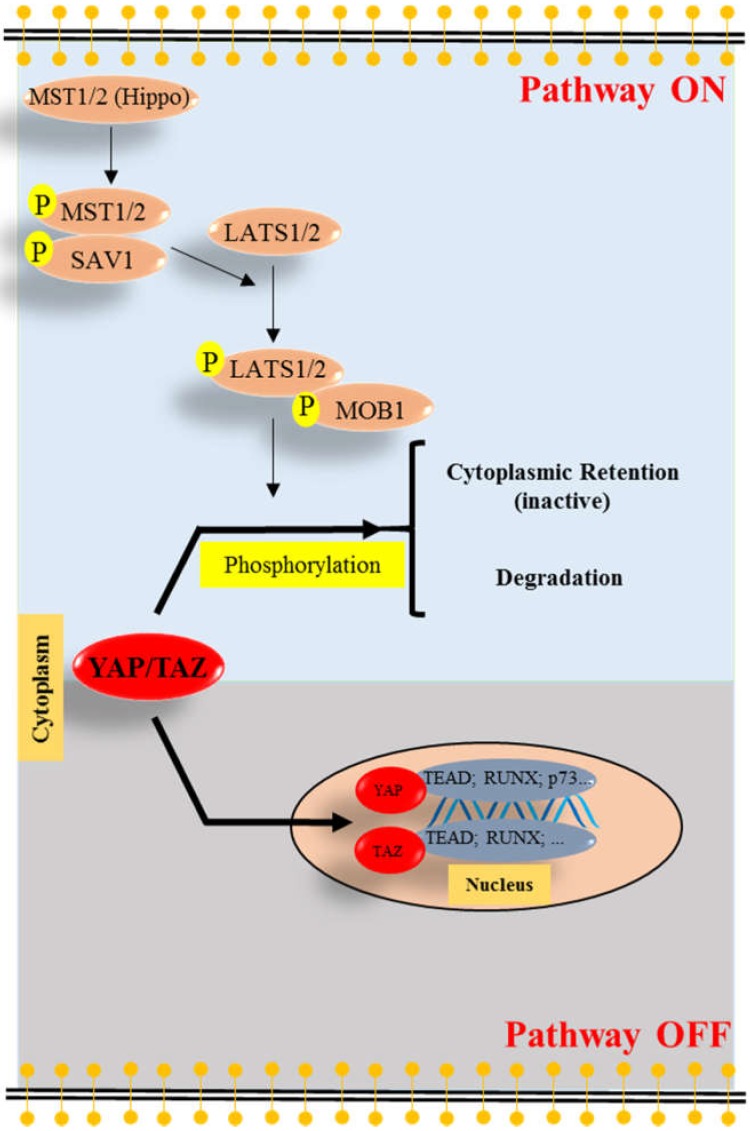
Components of the Hippo-YAP signaling pathway. When the pathway is switched “ON” (blue background), activated mammalian sterile 20-like kinases (MST1 and MST2, MST1/2) and Salvador homolog 1 (SAV1) phosphorylate and activate the large tumor suppressor kinases (LATS1 and LATS2, LATS1/2). The activated LATS1/2 and MOB kinase activators 1 (MOB1) phosphorylate yes-associated protein (YAP) or the transcriptional coactivator with PDZ-binding motif (TAZ), leading to YAP/TAZ cytoplasmic retention (inactive) and degradation. When the pathway is switched “OFF” (gray background), YAP/TAZ accumulates in the nucleus and forms complexes with some transcription factors such as TEA domain (TEAD) family transcription factors, runt-related transcription factors (RUNX) and p73. Arrow: Increase.

Yes-associated protein (YAP) is a core component of the Hippo signaling pathway in mammals [1]. Initially, this pathway was described to control organ size [2]. However, recently it has been discovered that YAP is also involved in oncogenesis [3,4] as well as apoptosis [5,6,7,8,9,10,11]. Hence, YAP is considered to be an emerging target to treat cancer. In mammals, multiple proteins such as the mammalian sterile 20-like kinases (MST1 and MST2, MST1/2), the large tumor suppressor kinases (LATS1 and LATS2, LATS1/2), the yes-associated protein (YAP), the transcriptional coactivator with PDZ-binding motif (TAZ), and the TEA domain family (TEAD1-4) transcription factors are important components of the Hippo signaling pathway (Figure 1) [1,2,4]. In addition, other transcription factors such as runt-related transcription factors (RUNX) and p73 are also involved in this pathway [6,12].

It has been reported that, for example, high cellular density or stimulation of G-protein-coupled receptors can switch “ON” Hippo signaling pathway by phosphorylating MST1/2 [13,14]. Subsequently, LATS1/2 and YAP are phosphorylated, leading to YAP cytoplasmic retention (inactive) and degradation [13,14]. In contrast, when cells are grown under conditions of low density, this pathway is switched “OFF”, YAP can translocate to the nucleus [1,2,4,13]. Subsequently, it interacts with TEAD transcription factors and induces the expression of several genes, such as *Cyclooxygenase-2* (*COX-2*) [15], *BIRC 5* (*Survivin*) [16,17], *glucose-transporter 1* (*Glut1*) [18], and *glucose-transporter 3* (*Glut3*) [19].

However, it also has been found that when cells suffer DNA damage stress, nuclear YAP can interacts with p73 and enhances the transcription of pro-apoptotic genes, such as *p53AIP1* [5], *Bax* [6,20], *DR5* [7], and *PUMA* [8]. Consistent with this bivalent effect on apoptosis, the role of YAP in cancer is also contradictive. For example, several studies demonstrated that YAP is highly expressed in pancreatic cancer and that high expression correlates with poor survival [21,22]. In addition, recent evidence proved that YAP promotes metastasis and proliferation of breast cancer cells [23,24] and contributes to the poor metastasis-free survival of these patients [25]. Thus, these studies suggest that YAP is involved in oncogenesis. However, Yuan et al. found that YAP is barely expressed in human breast cancer tissue and can be considered to be a tumor suppressor [26]. Therefore, it seems to be context dependent, if YAP can serve as a tumor suppressor or an oncogene.

Thus, in this review, we summarized the clinical relevance of YAP for cancer pathophysiology. We also reviewed molecules, processes and drugs, which are involved in Hippo-YAP signaling and their effect on apoptosis. Finally, we defined issues which should be addressed in the future.

## 2. The Anti-Apoptotic Function of YAP

### 2.1. YAP Is Overexpressed in Cancer and Inhibits Apoptosis

Most clinical studies have demonstrated that YAP is overexpressed in tumors and associated with poor survival of patients with solid tumors [21,22,27], such as lung tumors [28], pancreatic tumors [21,22,27], and colorectal tumors [29]. In addition, several studies proved that the *YAP* gene is amplified in cervical, ovarian, and fallopian tube cancers [30,31]. Moreover, silencing the expression of *YAP* gene by shRNA [18,32,33] or siRNA [15,34,35,36,37,38,39,40,41,42,43,44,45,46,47,48] could induce apoptosis (Table 1). Consistent with this finding, overexpressing YAP significantly inhibited apoptosis of liver [38,49,50], pancreas [39], colorectal cancer [15,51], and lung cancer cells [52]. All these publications suggest that YAP inhibits apoptosis. This could lead to accelerated tumor growth, which might then cause poor survival of patients [53]. Thus, YAP has a pro-oncogenic function. However, Liu et al. found that *YAP* gene silencing failed to promote cell apoptosis in thyroid papillary carcinoma cells [54]. In addition, they demonstrated that *YAP* gene silencing inhibited c-Myc expression. Possibly the repression of the pro-apoptotic gene c-Myc prevented the induction of apoptosis in these cells [55]. This might explain why silencing the *YAP* gene failed to induce apoptosis in this study [54].

### 2.2. YAP Inhibits Apoptosis by Interacting with TEAD Transcription Factors

**Figure 2:**
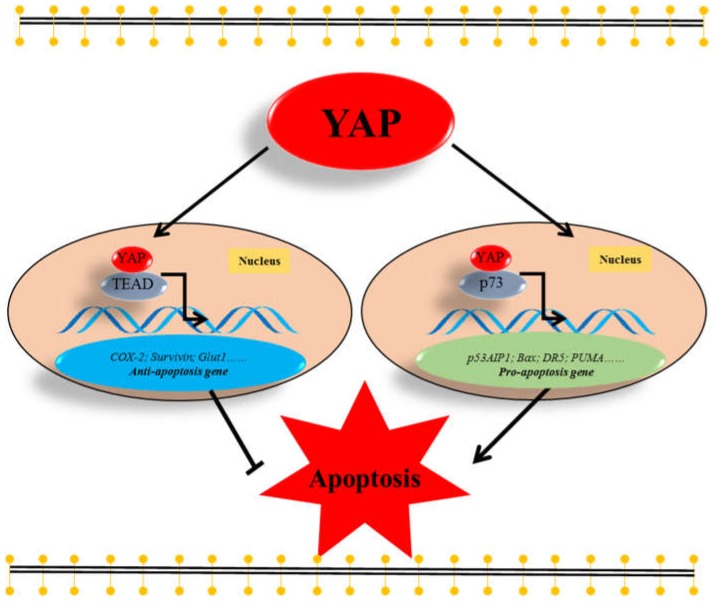
The bivalent role of YAP in apoptosis. In the nucleus, yes-associated protein (YAP) interacts with TEA domain (TEAD) family transcription factors and initiates the expression of anti-apoptotic genes, such as Cyclooxygenase-2 (COX-2), Survivin, and Glut1, to inhibit apoptosis. However, nuclear YAP can also interact with p73 to enhance the transcription of pro-apoptotic genes, such as p53AIP1, Bax, DR5, and PUMA to promote apoptosis. T bar: Inhibition. Arrow: Increase.

As a transcriptional co-activator, YAP does not contain a DNA-binding domain [4]. Thus, it requires binding to transcription factors of the TEAD family, TEAD1-4 [56,57], to stimulate anti-apoptotic gene expression (Figure 2). It has been demonstrated that YAP interacting with TEAD transcription factors could increase the expression of anti-apoptotic genes, such as *COX-2* [15], *Survivin* [16,17], and *Glut1* [18]. However, TEAD transcription factors are not the sole transcription factors, which bind to YAP. It has been reported that p73 is also a transcriptional partner of YAP and promotes the expression of several pro-apoptotic genes such as *p53AIP1* [5], *Bax* [6,20], *DR5* [7], and *PUMA* [8] (Figure 2). Thus, YAP can stimulate the expression of anti- as well as pro-apoptotic genes. This depends on the transcriptional partner of YAP (Figure 2).

### 2.3. YAP Inhibits Apoptosis by Increasing Glycolysis

Interestingly, Wang et al. demonstrated that glucose starvation can cause YAP phosphorylation at serine 127 and inhibits YAP transcriptional activity in human embryonic kidney 293T (HEK 293T) cells and cervical cancer cells [19]. Consistent with these findings, Lin et al. observed that knockdown of YAP significantly promoted cells apoptosis, when breast cancer cells were cultured at high glucose concentration [18]. However, when the cells were cultured at low glucose concentration, the anti-apoptotic role of YAP was largely abolished [18]. In addition, Wang et al. proved that YAP promotes the expression of *Glut3*, which is involved in glucose metabolism [19]. These data suggest that glucose starvation is an activator of Hippo-YAP signaling pathway, and that YAP inhibits apoptosis via regulating the uptake of glucose.

### 2.4. YAP Inhibits Apoptosis via Enhancing the Autophagic Flux

It has been reported that YAP decreases cisplatin-induced apoptosis through activation of autophagy in ovarian cancer cells [58]. Moreover, Yan et al. reported that knockdown of YAP causes apoptosis via reducing mitophagy, a selective degradation of mitochondria by autophagy, in gastric cancer cells [59]. In addition, Song et al. demonstrated that YAP enhanced the autophagic flux to reduce apoptosis in nutrient deprived breast cancer cells [60]. These publications are consistent with the concept that YAP inhibits apoptosis via inducing autophagic flux. However, Liu et al. found that inhibition of YAP accumulation failed to have an effect on apoptosis; but can induce autophagy in thyroid papillary carcinoma cells [54]. This indicates that even though most publications support the hypothesis that YAP inhibits apoptosis by increasing the autophagic flux, the YAP-induced autophagy is not always the sole or most important factor to regulate apoptosis in cancer cells.

## 3. The Pro-Apoptotic Function of YAP

### 3.1. Clinical Evidence Suggests That YAP Is a Context Specific Tumor Suppressor

Several studies demonstrated that YAP promotes apoptosis in vitro and therefore can potentially reduce tumor growth in vivo [5,6,7,8]. This implies that YAP can have an anti-oncogenic function. Indeed, some clinical evidence supports the concept that YAP is a tumor suppressor in breast cancer and hematological cancer [9,26]. Yuan et al. described that 63% of infiltrating ductal breast carcinomas had lost YAP expression, but they did not demonstrate that the decreased YAP expression was associated with a poor survival in breast cancer patients [26]. Cottini et al. found that low expression of YAP was associated with short survival time in hematological cancer [9]. However, this conclusion was based only on the analysis of YAP mRNA. Interestingly, this study also showed that upregulation of YAP expression induces cell death in an ABL1 (Abelson murine leukemia viral oncogene homolog 1) activity dependent manner [9]. Thus, these publications suggest that YAP can be a tumor suppressor in a context specific manner.

### 3.2. YAP Promotes Apoptosis in a p73-Dependent Manner

It has been demonstrated that nuclear YAP interacts with p73, a tumor suppressor, to enhance apoptosis in response to DNA-damage [6,61] (Figure 2). In addition, Levy et al. reported when normal hematological cells suffer DNA damage stress, tyrosine kinase c-Abl (ABL1) enters the nucleus and phosphorylates YAP on a tyrosine residue, Y357 [62]. The Y357-phosphorylated YAP binds to p73 to promote the transcription of pro-apoptotic genes, such as *p53AIPI* [5], *Bax* [6,20], *DR5* [7], and *PUMA* [8]. In addition, p300, which can activate p73, and promyelocytic leukemia (PML), which can increase YAP stabilization, are also involved in regulating this effect of the YAP-p73 complex [6,61]. These publications demonstrate that YAP can promote apoptosis via interaction with the transription factor p73.

## 4. A Multitude of Molecules Regulate Apoptosis and Hippo-YAP Signaling

**Figure 3:**
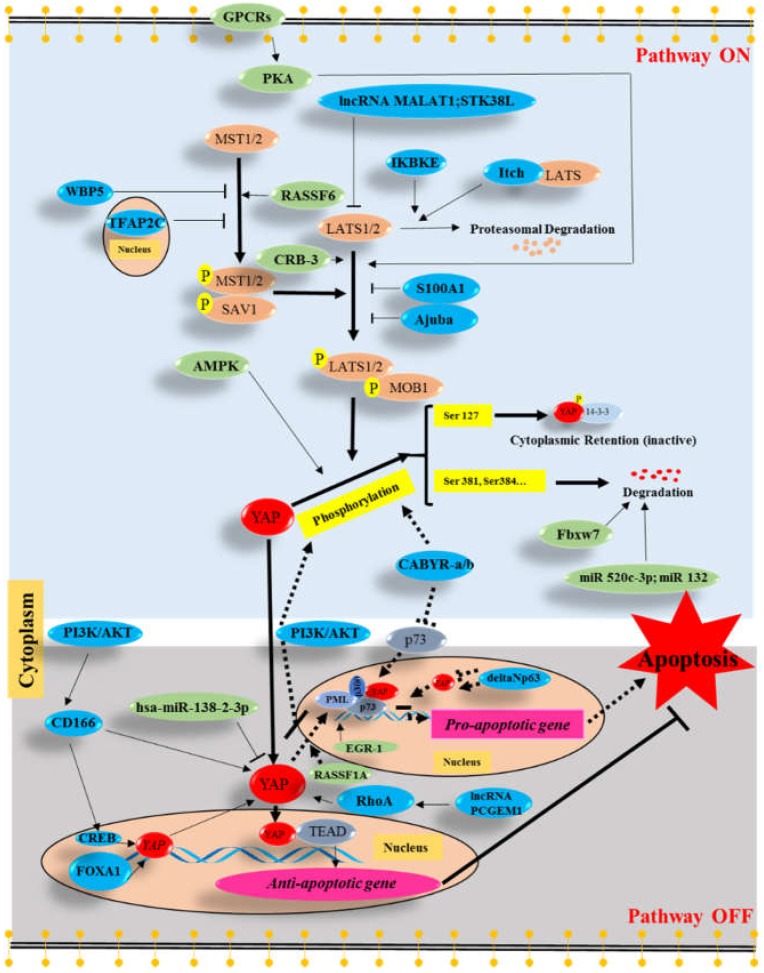
Many molecules regulate apoptosis and Hippo-YAP signaling pathway. Anti-apoptotic molecules are indicated in blue, whereas pro-apoptotic molecules are indicated in green. Most anti-apoptotic molecules, such as metastasis associated lung adenocarcinoma transcript 1 (MALAT1), serine/threonine kinase 38 like (STK38L), inhibitor of nuclear factor kappa B kinase subunit epsilon (IKBKE), Itch, S100 calcium-binding protein A1 (S100 A1), Ajuba, transcription factor AP-2 Gamma (TFAP2C), WW domain binding protein 5 (WBP5), lncRNA PCGEM1, Ras homolog gene family, member A (RhoA), usually allow YAP to enter the nucleus to interact with TEA domain (TEAD) family transcription factors and to activate the expression of anti-apoptotic genes. Some molecules, such as cAMP response element-binding protein (CREB) and forkhead box protein A1 (FoxA1) inhibit cell apoptosis by inducing the transcription of the *YAP* gene. In addition, deltaNp63 can suppress *YAP* gene expression to impair p73-mediated apoptosis. However, the role of deltaNp63 in *YAP* gene expression is still a matter of controversy. Moreover, Calcium-binding tyrosine phosphorylation-regulated protein (CABYR-a and CABYR-b, CABYR-a/b) and phosphatidylinositide 3-kinase (PI3K)/protein kinase B (AKT) signaling lead to phosphorylation of YAP at serine 127 and attenuate nuclear YAP/p73-mediated apoptosis. Additionally, PI3K/AKT signaling can also suppress apoptosis by upregulate CD166 expression. Most of the pro-apoptotic molecules, such as G-protein-coupled receptors (GPCRs), cAMP-dependent protein kinase A (PKA), Crumbs-3 (CRB-3), Ras association domain family member 6 (RASSF6), AMP-activation protein kinase (AMPK), enhance apoptosis by inducing phosphorylation of YAP and thereby impairing nuclear accumulation of YAP. In addition, some pro-apoptotic molecules, such as Fbxw7, miR520c-3p, miR132, and hsa-miR-138-2-3p enhance the degradation or block the expression of YAP. In addition, RAS association domain family 1 isoform A (RASSF1A) and early growth response-1 (EGR-1) can induce pro-apoptotic genes via the YAP-p73-p300-PML complex. T bar: Inhibition. Arrow: Increase.

Molecules, which inhibit or induce apoptosis and regulate Hippo-YAP signaling, were summarized in Figure 3 and Table 2. In the nucleus, YAP often interacts with TEAD transcription factors to stimulate the expression of anti-apoptotic genes [15,18,44]. However, it can also bind to p73 and promote the expression of pro-apoptotic genes [5,6,7,8,20].

### 4.1. Activators of YAP, Which Impede Apoptosis

#### 4.1.1. TFAP2C

The transcription factor AP-2 Gamma (TFAP2C) is a member of the activating protein 2 (AP-2) family [63]. Some studies demonstrate that TFAP2C can increase the accumulation of cellular YAP in the nucleus by inactivating MST1/2 [42,64]. Moreover, Wang et al. reported that overexpressed TFAP2C could decrease the 5-fluorouracil-induced apoptosis in colorectal cancer cells [42]. Interestingly, they observed that this effect of TFAP2C is dependent on Rho-associated protein kinase (ROCK). This suggests that TRAFP2C inhibits apoptosis via YAP and ROCK signaling. Indeed, another publication describes that the ROCK inhibitor, Y-27632, blocked the nuclear accumulation of YAP. This demonstrates that ROCK signaling indeed regulates YAP [65]. These data imply that TFAP2C inhibits cell apoptosis via ROCK-YAP signaling and suggest an anti-apoptotic function of YAP.

#### 4.1.2. WBP5

Initially, WW domain binding protein 5 (WBP5) was described as a ligand that can bind to FBP11 WW domain [66]. Recently, it was reported that WBP5 was involved in regulating the Hippo pathway [67]. Tang et al. demonstrated that overexpressed WBP5 could inhibit the phosphorylation of MST2 and YAP without changing the level of these two proteins in lung cancer cells [68]. Moreover, immunofluorescence assays proved that upregulation of WBP5 induced the nuclear accumulation of YAP and decreased apoptosis, whereas downregulation of WBP5 lead to YAP inactive and enhanced cell apoptosis. These studies suggest an anti-apoptotic function of YAP and that WBP5 activates YAP signaling and inhibits apoptosis.

#### 4.1.3. lncRNA MALAT1 and STK38L

It has been reported that downregulation of lncRNA metastasis associated lung adenocarcinoma transcript 1 (MALAT1) and serine/threonine kinase 38 like (STK38L) lead to the accumulation of LATS1 and decrease the level of cellular YAP in pancreatic cancer cells [69,70]. However, the mechanism, how lncRNA MALAT1 and STK38L regulate the accumulation of LATS1 and YAP, is still unknown. In addition, these studies also demonstrated that lncRNA MALAT1 and STK38L could inhibit apoptosis via unknown mechanisms [69,70]. These anti-apoptotic effects may be mediated by YAP and suggest an anti-apoptotic function of YAP.

#### 4.1.4. IKBKE and Itch

Liu et al. demonstrated that the knockdown of nuclear factor kappa B kinase subunit epsilon (IKBKE) dramatically elevated LATS1/2 concentration and serine 127-phosphorylated YAP in human glioblastoma cells [71]. In contrast, it decreased the nuclear localization of YAP [71]. Moreover, inhibition of IKBKE by amlexanox suppressed the accumulation of cellular YAP and the anti-apoptotic protein, CYR61 [71,72]. These data imply that IKBKE may inhibit apoptosis by inducing the nuclear localization of YAP. This suggests a pro-oncogenic activity of YAP [71].

In addition, it was reported that Itch, a HECT class E3 ubiquitin ligase, could complex with LATS1 through the WW domains of Itch and the PPxY motifs of LATS1 [73]. Ho et al. reported that downregulation of Itch not only provoked the stabilization of LATS1, but also induced phosphorylation of YAP at serine 127 in HEK 293T cells [73]. In addition, downregulation of Itch induced cell death, while overexpressed Itch yielded the opposite effect. Thus, these data suggest that Itch can inhibit the phosphorylation of YAP and reduces apoptosis via enhancing LATS1 degradation. In addition, these data suggest an anti-apoptotic activity of YAP.

#### 4.1.5. S100 A1

Recently, Guo et al. demonstrated that S100 calcium-binding protein A1 (S100 A1), which interacts with LATS1, inhibits the phosphorylation of this kinase and leads to decreased phosphorylation and increased accumulation of cellular YAP [74]. The authors also demonstrated that knockdown of S100A1 by siRNA increased cisplatin-induced apoptosis. In addition, LATS1 depletion significantly reduced the effects of S100A1 on apoptosis. Thus, this suggests that S100 A1 inhibits apoptosis and increases the accumulation of YAP. This argues for an anti-apoptotic function of YAP.

#### 4.1.6. Ajuba

It was reported that Ajuba, an actin binding and scaffolding protein, can interact with LATS1/2 and thereby inhibits the activation of this kinase [75]. This leads to decreased YAP phosphorylation [75]. Interestingly, Ajuba does not only decrease the phosphorylation of YAP, but also inhibits apoptosis of cervical cancer cells [76]. This suggests that inhibition of apoptosis by Ajuba correlates with decreased YAP phosphorylation and implies an anti-apoptotic function of YAP. However, we do not fully understand if and how YAP signaling is important for the inhibition of apoptosis by Ajuba.

#### 4.1.7. lncRNA PCGEM1 and RhoA

Prostate cancer gene expression marker 1 (PCGEM1) is an lncRNA that is initially found to be overexpressed in aggressive prostate cancers [77]. Recently, Chen et al. reported that overexpressed lncRNA PCGEM1 could decrease apoptosis in ovarian cancer cells [78]. Subsequently, they found that upregulated lncRNA PCGEM1 increased the expression of RhoA, which can enhance activity and accumulation of cellular YAP [79]. In addition, the authors also reported that downregulated PCGEM1 could promote apoptosis via decreasing RhoA expression. However, silencing the expression of RhoA reversed the anti-apoptotic effect of PCGEM1 and significantly inhibited the total level of YAP protein [78]. This suggests that PCGEM1 inhibits apoptosis and induces the accumulation of YAP via RhoA. Thus, YAP might have an anti-apoptotic function in this context.

#### 4.1.8. CREB and FOXA1

Cyclic adenosine monophosphate (cAMP) response element-binding (CREB) protein is a ubiquitous transcription factor that activates the transcriptional activity of various promoters [80]. Wang et al. reported that CREB could promote YAP transcription through binding to a novel region (608/439 base pairs) within the *YAP* promoter in liver cancer cells [81]. In addition, the same research group also found that CD166 could inhibit apoptosis via increasing the accumulation of CREB and cellular YAP [49]. These studies suggest that YAP might be anti-apoptotic.

Some studies demonstrated that forkhead box protein A1 (FOXA1), a member of forkhead box gene superfamily, inhibits apoptosis in cancer cells [82,83]. Consistent with these in vitro data, Ren et al. observed that gastric cancer patients with high expression of FOXA1 had poorer five-year overall survival [84]. Moreover, Ma et al. reported that FOXA1 could be detected in 57.8% (52/90) of the colorectal cancer specimens, whereas only in 37.8% (34/90) of the non-cancerous specimens [85]. Moreover, the patients with FOXA1 expression had poor survival. It was also demonstrated that FOXA1 knockdown evidently induced apoptosis; while it decreased the expression of *YAP* [85]. Interestingly, Yu et al. reported that in liver cancer cells FOXA1 was able to bind the R2 region of the *YAP* promoter, which contains a CREB binding motif [86]. Additionally, FOXA1 overexpression recruited CREB onto the R2 region. These data suggest that FOXA1 facilitates *YAP* transcription via enhancing the binding of CREB to the *YAP* promoter. These studies demonstrate that FOXA1 inhibits apoptosis and increases the expression of *YAP*, which suggests an anti-apoptotic function of YAP.

### 4.2. Activators of YAP, Which Induce Apoptosis

#### 4.2.1. RASSF1A

Numerous studies have argued that RASSF1A (Ras association domain family 1 isoform A) is a tumor suppressor [8,87,88]. Matallanas et al. found that RASSF1A allows YAP to move to the nucleus and to interact with p73 [8]. The YAP-p73 complex results in transcription of the pro-apoptotic target gene *PUMA* in breast cancer cells [8] and *Ankyrin Repeat Domain 1* (*ANKRD1*), which is in some circumstances considered to be a tumor suppressor gene, because it is epigenetically inactivated in human cancer and reduces colony formation of cancer cells [87]. In addition, Yee et al. found that a RASSF1A polymorphism, RASSF1A-p.133Ser, failed to enhance YAP-p73 mediated apoptosis [88]. Furthermore, they demonstrated that male soft tissue sarcoma patients, who carried the RASSF1A-p.133Ser allele, exhibited poorer tumor-specific survival [88]. This suggests that the tumor suppressor function of RASSF1A may be due to YAP-p73 mediated apoptosis. However, Donninger et al. reported that the anti-tumor function of RASSF1 is rather caused by inhibition of proliferation than its pro-apoptotic effect [89]. Nevertheless, all these publications suggest that RASSF1A induces apoptosis and that YAP can have a pro-apoptotic function, when interacting with p73.

#### 4.2.2. EGR-1

Early growth response-1 (EGR-1) is a nuclear protein and functions as a transcriptional regulator [90]. Zagurovskaya et al. demonstrated that EGR-1 interacts with YAP through its PPxY motif and that this interaction is required for inducing clonogenic cell death in prostate carcinoma cells [91]. In addition, several studies reported that EGR-1 served as a tumor suppressor in colorectal carcinoma [92,93,94], gliomas [92,93,94], and colon carcinoma [92,93,94]. However, the role of EGR-1 in cancer is still a matter of controversy [95]. For example, Virolle et al. proved that EGR1 promotes the progression of prostate cancer [96]. These studies suggest that EGR-1 induces cell death and YAP might have a pro-apoptotic function when interacting with EGR-1.

### 4.3. Inhibitors of YAP, Which Induce Apoptosis

#### 4.3.1. GPCRs and PKA

G-protein-coupled receptors (GPCRs), the largest family of cell surface receptors, have been considered to be upstream regulators of the Hippo pathway [14,97,98]. Yu et al. reported that lysophosphatidic acid (LPA) and sphingosine 1-phosphophate (S1-P) inhibit LATS1/2 activity via blocking G12- and G13-coupled receptors, which activate YAP function and promote cell migration and proliferation [14]. In contrast, GPCRs activators, such as glucagon and epinephrine could activate LATS1/2 and inhibit YAP function [14]. Kim et al. observed that the GPRCs-triggered LATS2-YAP pathway depends on cAMP-dependent protein kinase A (PKA) [97]. In addition, they demonstrated that PKA could phosphorylate LATS2, and thereby enhances LATS2 activity sufficiently to phosphorylate YAP at serine 381 [97], which leads to the degradation of YAP [99]. Moreover, Zhang et al. also observed that GPCRs and PKA could induce YAP phosphorylation via promoting LATS1 phosphorylation [98]. Consistent with these findings, the authors reported that induction of apoptosis by omega-3 polyunsaturated fatty acid is mediated by GPCRs [98]. These data suggest that GPCR/PKA signaling induces apoptosis and inhibits YAP function, suggesting an anti-apoptotic function of YAP.

#### 4.3.2. RASSF6

He et al. found that Ras association domain family member 6 (RASSF6) overexpression could increase cisplatin-induced apoptosis, while depletion of RASSF6 had the opposite effect in breast cancer cells [100]. The authors observed that RASSF6 decreased cellular YAP concentration and activated the Hippo signaling pathway by upregulating the phosphorylation of MST1/2 and LATS1. In addition, overexpression of YAP inhibited the RASSF6 and cisplatin-induced apoptosis. Thus, these data suggest that RASSF6 induces apoptosis through activation of the Hippo pathway leading to the inhibition of YAP function. These data suggest an anti-apoptotic function of YAP.

#### 4.3.3. CRB-3

Recently, it was reported that crumbs-3 (CRB-3), the major crumbs isoform in mammalian epithelial cells [101], is involved in Hippo signaling. Mao et al. found that overexpressed CRB-3 could induce the serine 127-phosphorylated YAP, and decreased the accumulation of nuclear YAP protein in mammary epithelial cells [102]. In addition, they also reported in a xenograft study that CRB-3 increased cell death within tumors. Moreover, Szymaniak et al. demonstrated that CRB-3 promoted the interaction between YAP and LATS1/2 in lung epithelial cells [103]. This led to increased phosphorylation and cytoplasmic sequestration of YAP [103]. Thus, CRB-3 blocks YAP function and induces cell death, implying a pro-oncogenic function of YAP.

#### 4.3.4. AMPK

Mo et al. reported that the activation of AMP-activation protein kinase (AMPK) inhibits the activity of YAP [104]. They demonstrated that metformin or aminoimidazole carboxamide ribonucleotide (AICAR), two well-known AMPK activators, increased the serine 127-phosphorylated YAP in primary mouse hepatocytes. Moreover, these two substances decreased the expression of anti-apoptotic genes, such as *CTGF* and *CYR61* [72,105]. Consistent with this study, Wang et al. also found that an AMPKα1 *C*-terminal-truncated mutant (amino acids 1-312), which has been demonstrated to be a constitutively active form of AMPK, induced the phosphorylation of YAP in HEK 293T cells [19]. In addition, Jiang et al. proved that resveratrol, a natural polyphenol present in most plants, inhibited YAP accumulation in pancreatic cancer cells [40]. However, knockdown of AMPK rescued the resveratrol-induced suppression of YAP [40]. This suggests that AMPK induces apoptosis and impairs the function of YAP via enhancing YAP phosphorylation. This could lead in vivo to larger tumors, suggesting a pro-oncogenic function of YAP.

#### 4.3.5. Fbxw7, miR520c-3p, miR132, and hsa-miR-138-2-3p

It was suggested that F-box and WD repeat domain-containing 7 (Fbxw7), a well-known F-box protein in the SCF (SKP1-CUL1-F-box protein) E3 ligase complex [106], can directly bind to YAP and decreases the accumulation of YAP in hepatocellular carcinoma cells [107]. In addition, the proteasome inhibitor MG132 was able to prevent the downregulation of YAP in Fbxw7 overexpressing cells, suggesting that Fbxw7 targets YAP for degradation by proteasomes [107]. Tu et al. reported that Fbxw7 expression could induce cell apoptosis and that restoring YAP expression lead to a significant reduction of apoptosis [107]. Thus, these data suggest that Fbxw7 induces apoptosis by inducing the degradation of YAP. Since loss of Fbxw7 expression was associated with poor clinicopathological features including large tumor size [107], YAP might have a pro-oncogenic function in this context.

Some molecules, such as miR520c-3p [108] or miR132 [108], have been reported to enhance the degradation of YAP and induce apoptosis of hepatocellular carcinoma cells by an unknown mechanisms. In addition, Zhu et al. reported that hsa-miR-138-2-3p prevented the accumulation of cellular YAP and induced apoptosis in human laryngeal cancer stem cells [109]. These publications also argue for an anti-apoptotic function of YAP.

### 4.4. Inhibitors of YAP Which Impede Apoptosis

#### 4.4.1. DeltaNp63

DeltaNp63 is an isoform of p63, which lacks the acidic transactivation (TA) domain and antagonizes p53, TAp63 and TAp73 by inhibiting the expression of their downstream target genes [110]. Interestingly, Ehsanian et al. observed that deltaNp63 binds to the promotor of *YAP* and suppresses *YAP* expression in head and neck cancer cells [111]. Surprisingly, Li et al. also observed that deltaNp63 directly binds to the YAP promoter, but claimed that this induces *YAP* gene expression in squamous cell carcinoma cell lines [112]. Possibly, the effect of deltaNp63 on gene expression of target genes depends on many additional transcription factors, which might be differently expressed in distinct cell lines. In addition, Ehsanian et al. could also clearly demonstrate that deltaNp63 and the knock down of YAP inhibits apoptosis [111]. This suggests that YAP has a pro-apoptotic effect in head and neck cancer cells.

#### 4.4.2. CABYR

Calcium-binding tyrosine phosphorylation-regulated (CABYR) protein is isolated from human spermatozoa and participates in the sperm capacitation [113]. Recently, Xiao et al. described that, in lung cancer cells, silencing of the *CABYR-a* and *CABYR-b* genes (*CABYR-a/b*) inhibits the phosphorylation of YAP at serine 127, which usually leads to increased nuclear localization of YAP [7]. This study also demonstrated that silencing of *CABYR-a/b* increased the percentage of dead cells and that this induction of apoptosis could be inhibited by knocking down of YAP and p73 [7]. Moreover, overexpression of YAP plus p73, but not the expression of either protein alone, effectively promoted cell apoptosis, suggesting that both proteins must be present to induce apoptosis. These data suggest that depletion of CABYR-a/b sensitizes lung cancer cells to apoptosis in a YAP/p73 dependent manner [7]. This implies that YAP, when interacting with p73, has a pro-apoptotic effect in lung cancer cells.

#### 4.4.3. PI3K/AKT

Current evidence demonstrates that activated phosphatidylinositide 3-kinase (PI3K)/protein kinase B (AKT) signaling can phosphorylate YAP at serine 127 [114] (Table 2). The serine 127-phosphorylated YAP interacts with 14-3-3 and is sequestered in the cytoplasm, which attenuates nuclear YAP/p73-mediated apoptosis [114]. Consistent with this publication, Ehsanian et al. could also demonstrate that AKT can inhibit apoptosis via phosphorylating YAP at serine 127 [111]. In addition, PI3K/AKT signaling can also upregulate CD166 expression [49]. Subsequently, CD166 enhances YAP expression and activity to suppress apoptosis in liver cancer cells [49]. These data suggest that PI3K/AKT signaling inhibits apoptosis by regulating YAP activity via at least two different mechanisms.

## 5. Compounds, Regulating Hippo-YAP Signaling, Induce Apoptosis, and Impair Cancer

Some studies demonstrate that Hippo-YAP signaling might be a promising target for therapies to impair cancer [115]. Since YAP is the most important functional component of the Hippo-YAP signaling pathway, it may be a more promising therapeutic target than other proteins.

Several compounds have been proven to regulate apoptosis via, or partly via, regulating Hippo-YAP signaling (Table 3). These compounds can be classified into five categories: (a) compounds that regulate upstream molecules of YAP or YAP per se to inhibit YAP accumulation, such as omega-3 polyunsaturated fatty acids (ω-3 PUFAs) [98], gossypol [116], resveratrol [40], 17-DMAG [117], amlexanox [71] and tubacin [117], norcantharidin [118,119], JQ1 [120], oligomeric proanthocyanidins (OPCs) [121]; (b) compounds that promote the phosphorylation of YAP and block YAP nuclear translocation, such as dobutamine [122], huaier [123], GCCSysm-4 (G4) [15], scutellarin [124] and hydrogen sulfide-releasing oleanolic acid (HS-OA) [125]; (c) compounds that inhibit the interaction of YAP and TEAD transcription factors, such as verteporfin [126,127,128] and CA3 [129], or inhibit the interaction of YAP and p63, such as nicotine [130]; (d) compounds that increase YAP accumulation, such as IBS003031 [131] and actinomycin D [132]; (e) compounds that regulate the YAP-p73 complex, such as α-TEA [133]. Many of these mentioned compounds are currently used in clinical trials (Table 3). This increases the expectation that targeting the Hippo-YAP signaling pathway will become an efficient way to treat cancer.

## 6. Conclusions and Future Perspectives

In conclusion, current evidence suggests that YAP, the core component of Hippo-YAP signaling pathway, has an ambivalent role in cell apoptosis. It can bind to TEAD transcription factors to promote the transcription of anti-apoptotic genes, such as *COX-2* [15], *Survivin* [16,17], and *Glut1* [18]. However, it can also initiate the transcription of pro-apoptotic genes, such as *p53AIP1* [5], *Bax* [6,20], *DR5* [7], and *PUMA* [8]. In addition, the clinical data also demonstrate that YAP can function as an oncogene in several cancers [21,22,27], while it can also serve as a tumor suppressor in breast cancer [60] and hematological cancer [9]. This suggests YAP has ambivalent functions. It can promote or inhibit tumor progression dependent on other signaling pathways and the cancer type. These data also imply that a therapy, which targets the Hippo-YAP signaling pathway, might be of benefit to only a subset of patients.

Thus, there are several questions that should be addressed before targeting Hippo-YAP signaling pathway to treat cancer patients: One main question is, if YAP inhibitors in combination with traditional drugs, such as gemcitabine and cisplatin, induce cell death and impair cancer growth. Several promising studies have already demonstrated that YAP inhibitors can restore sensitivity to gemcitabine and cisplatin in several cancers [40,134,135]. On the contrary, Gujral et al. described that nuclear YAP enhances gemcitabine effectiveness by downregulating multidrug transporters [136]. In addition, previous studies also found that YAP enhances p73 mediated apoptosis when DNA damage stress is induced by cisplatin [5,6,62]. These contradictory studies suggest that YAP inhibitors might not always inhibit tumor growth, but might also foster tumor growth. Other important questions are: (a) Is nuclear YAP abnormally expressed in individual cancer types and is its expression associated with poor survival or good survival of cancer patients? This is crucial for deciding, if a YAP inhibitor or a YAP activator should be applied. (b) How do distinct drugs, which regulate Hippo-YAP signaling, compare in their efficacy to each other? (c) Are some drugs especially useful, because they do not only regulate YAP, but also modify other signaling pathways? These questions need to be addressed in order to provide a solid basis for planning clinical trials.

## 7. Note

This review was performed according to the PRISMA guidelines. Publications were identified by searching PubMed, on 5 July 2018, using the following search strategy: (Hippo [tiab] OR YAP [tiab]) AND (apoptosis [tiab] OR autophagy [tiab]) AND (neoplasms [tiab] OR cancer [tiab]). Inclusion criteria: We included all studies which investigated the “mechanism of interaction between YAP and autophagy” or the “mechanisms of interaction between YAP and apoptosis”. Exclusion criteria: We excluded article types, which were reviews or commentaries; literature written not in English and irrelevant literature (literature which does not meet the inclusion criteria). We also added publications, which were cited in the reference list of the included literature or suggested by reviewers. In addition, we searched the ClinicalTrials.gov data base, in order to find clinical trials using drugs, which are involved in regulating the Hippo-YAP signaling pathway.

## Figures and Tables

**Table 1:** Manipulation of YAP expression and its effect on apoptosis.

Cancer	Method	YAP Expression	Apoptosis	PMID
Esophagus	shRNA	Decreased	Increased	27307755 [32]
Lung	shRNA			25665005 [33]
Breast	shRNA			28892790 [18]
Stomach	siRNA			27073556 [34]
Stomach	siRNA			21805037 [35]
Liver	siRNA			23419361 [36]
Liver	siRNA			27323827 [37]
Liver	siRNA			29928445 [38]
Pancreas	siRNA			27738325 [39]
Pancreas	siRNA			27669292 [40]
Pancreas	siRNA			22396793 [41]
Colon/Rectum	siRNA			29439714 [42]
Colon/Rectum	shRNA			29241219 [43]
Colon/Rectum	shRNA			29037225 [15]
Colon/Rectum	siRNA			26944315 [44]
Colon/Rectum	siRNA			29512779 [45]
Ovarian	siRNA			29848699 [46]
Prostate	siRNA			26126522 [47]
Rhabdomyosarcoma	shRNA			26496700 [48]
Lung	cDNA	Increased	Decreased	20219076 [52]
Liver	cDNA			24482231 [49]
Liver	cDNA			27359056 [50]
Liver	cDNA			29928445 [38]
Pancreas	cDNA			27738325 [39]
Colon/Rectum	cDNA			29037225 [15]
Colon/Rectum	cDNA			29042987 [51]
Thyroid	siRNA	Decreased	-	28804541 [54]

-: *YAP* gene silencing failed to promote cell apoptosis.

**Table 2:** Molecules regulate Hippo-YAP signaling and apoptosis.

Molecule	Target	YAP	Apoptosis	PMID
**a. Activators of YAP, which impede apoptosis**				
TFAP2C	MST1/2	Increases nuclear YAP	Decreased	29439714 [42]
WBP5	MST2	Increases nuclear YAP	Decreased	27336605 [68]
lncRNA MALAT1	LATS1	Increases cellular YAP	Decreased	29215734 [69]
STK38L	LATS2	Increases cellular YAP	Decreased	29108249 [70]
IKBKE	LATS1/2	Decreases phosphorylation of YAP	Decreased	29048430 [71]
Itch	LATS1	Decreases phosphorylation of YAP	Decreased	21383157 [73]
S100 A1	LATS1	Decreases phosphorylation of YAP	Decreased	29901195 [74]
Ajuba	LATS1/2	Decreases phosphorylation of YAP	Decreased	20303269 [75]
lncRNA PCGEM1	RhoA	Increases cellular YAP	Decreased	29949791 [78]
CREB	YAP	Increases *YAP* gene transcription	Decreased	24482231 [49]
**b. Activators of YAP, which induce apoptosis**				
RASSF1A	YAP	Increases nuclear YAP	Increased	17889669 [8]
EGR-1	YAP	Forms a complex with YAP	Increased	19137013 [91]
**c. Inhibitors of YAP, which induce apoptosis**				
GPCRs	PKA	Phosphorylates YAP at serine 381	Increased	23644383 [97]
RASSF6	MST1/2	Decreases cellular YAP	Increased	29964010 [100]
CRB-3	LATS1/2	Increases phosphorylation of YAP	Increased	28079891 [102]
AMPK	YAP	Increases phosphorylation of YAP	Increased	25751140 [104]
Fbxw7	YAP	Increases YAP degradation	Increased	24884509 [107]
miR520c-3p	YAP	Decreases *YAP* gene expression	Increased	27633306 [108]
miR132	YAP	Decreases *YAP* gene expression	Increased	27633306 [108]
Hsa-miR-138-2-3p	YAP	Decreases cellular YAP	Increased	28533948 [109]
**d. Inhibitors of YAP, which impede apoptosis**				
deltaNp63	YAP	Controversy in *YAP* gene expression	Decreased	28923839 [111]
CABYR	YAP	Increases phosphorylation of YAP	Decreased	26843620 [7]
PI3K/AKT	YAP	Increases phosphorylation of YAP	Decreased	12535517 [114]

Phosphorylates YAP at serine 127.

**Table 3:** Compounds regulating Hippo-YAP signaling, tumor progression, and apoptosis.

Compounds	Cancer	Target	TW/TV *	Apoptosis	Clinical Trials ^†^	PMID
**Regulate upstream molecules of YAP or YAP per se to decrease YAP expression**						
ω-3 PUFAs	CR	GPCRs	?	I	>20	27506947 [98]
Gossypol	Ovarian	LATS1	?	I	10	25180175 [116]
Resveratrol	Pancreas	AMPK	?	I	9	27669292 [40]
17-DMAG	Breast	HSP90	D	I	4	28529458 [117]
Amlexanox	Glioblastoma	IKBKE	D	I	1	29048430 [71]
Tubacin	Breast	HDAC6	D	I	0	28529458 [117]
Norcantharidin	Lung	YAP	?	I	0	29901163 [118] 27903989 [119]
JQ1	Chondrosarcoma	YAP	?	I	0	28059436 [120]
OPCs	CR	YAP	D	I	0	29463813 [121]
**Promote the phosphorylation of YAP and block YAP nuclear translocation**						
Dobutamine	Stomach	YAP	?	I	>20	25493021 [122]
Huaier	Liver	YAP	?	I	6	29187885 [123]
G4	CR	YAP	D	I	0	29037225 [15]
Scutellarin	Breast	YAP	D	I	0	29079722 [124]
HS-OA	Liver	YAP-14-3-3	D	I	0	27437776 [125]
**Inhibit the interaction of YAP and TEAD or the interaction of YAP and p63**						
Verteporfin	Pancreas	YAP-TEAD	D	I		28002618 [126]
	UM		?	I	9	28042502 [127]
	CR		?	I		27383277 [128]
CA3	Esophagus	YAP-TEAD	D	I	0	29167315 [129]
Nicotine	Esophagus	YAP-p63	?	D	>20	24621512 [130]
Increase YAP Expression						
IBS003031	MM	YAP	?	I	0	29061667 [131]
Actinomycin D	Liver	YAP	D	I	>20	27836738 [132]
**Regulate the YAP-p73 complex**						
α-TEA	Breast	YAP-p73	?	I	1	21214929 [133]

* TW: Tumor weight; TV: Tumor volume; D: Decreased; † search in https://clinicaltrials.gov/; we excluded clinical trial with status of not yet recruiting, suspended, terminated, withdrawn and unknown. ω-3 PUFAs: Omega-3 polyunsaturated fatty acids; CR: Colon/Rectum; GPCRs: G-protein-coupled receptors; LATS1: Large tumor suppressor kinases1; AMPK: AMP-activation protein kinase; HSP90: Heat shock protein 9; IKBKE: Inhibitor of nuclear factor kappa B kinase subunit epsilon; HDAC6: Histone deacetylase 6; OPCs: Oligomeric proanthocyanidins; G4: GCCSysm-4; HS-OA: Hydrogen sulfide-releasing oleanolic acid; UM: Uveal melanoma; MM: Multiple myeloma. ?: Studies did not measure tumor weight or tumor volume.
